# The CTRP3-AdipoR2 Axis Regulates the Development of Experimental Autoimmune Encephalomyelitis by Suppressing Th17 Cell Differentiation

**DOI:** 10.3389/fimmu.2021.607346

**Published:** 2021-12-02

**Authors:** Masanori A. Murayama, Hsi-Hua Chi, Mako Matsuoka, Takahiro Ono, Yoichiro Iwakura

**Affiliations:** ^1^ Center for Animal Disease Models, Research Institute for Biomedical Sciences, Tokyo University of Science, Chiba, Japan; ^2^ Department of Animal Models for Human Diseases, Institute of Biomedical Science, Kansai Medical University, Osaka, Japan

**Keywords:** CTRP3, Th17 cells, AdipoR2, EAE, autoimmune diseases

## Abstract

C1q/TNF-related proteins (CTRP) including CTRP3 are a group of secreted proteins which have a complement C1q-like domain in common, and play versatile roles in lipid metabolism, inflammation, tumor metastasis and bone metabolism. Previously, we showed that the expression of *C1qtnf3*, encoding CTRP3, is highly augmented in joints of autoimmune arthritis models and CTRP3-deficiency exacerbates collagen-induced arthritis in mice. However, the mechanisms how CTRP3-deficiency exacerbates arthritis still remain to be elucidated. In this study, we showed that CTRP3 was highly expressed in Th17 cell, a key player for the development of autoimmune diseases, and Th17 cell differentiation was augmented in *C1qtnf3^–/–^* mice. Th17 cell differentiation, but not Th1 cell differentiation, was suppressed by CTRP3 and this suppression was abolished by the treatment with a receptor antagonist against AdipoR2, but not AdipoR1, associated with suppression of *Rorc* and *Stat3* expression. Furthermore, AdipoR1 and AdipoR2 agonist, AdipoRon suppressed Th17 cell differentiation *via* AdipoR2, but not AdipoR1. The development of myelin oligodendrocyte glycoprotein (MOG)-induced experimental autoimmune encephalomyelitis was enhanced in *C1qtnf3* ^–/–^ mice associated with increase of Th17 cell population. CTRP3 inhibited MOG-induced IL-17 production from T cells by affecting both T cells and dendritic cells. These results show that CTRP3 is an endogenous regulator of Th17 differentiation, suggesting that the CTRP3-AdipoR2 axis is a good target for the treatment of Th17 cell-mediated diseases.

## Introduction

C1q/TNF-related protein (CTRP) superfamily consists of more than 30 family members including TNF, adiponectin, CTRP3 (also known as CORS26, cartducin, or cartnectin) and CTRP6, which have a complement C1q domain-like structure in common (1, 2). Many of CTRP family members are categorized as adipokines, because these CTRP family members are secreted from adipose tissues. Functionally, this superfamily was suggested to be involved in a wide range of physiological and pathological processes such as lipid metabolism, inflammation, tumor metastasis and bone metabolism (3).

Progestin and adipoQ receptor (PAQR) family members are the receptors for the CTRP family members, and PAQR1 (also known as AdipoR1), PAQR2 (AdipoR2) and PAQR3 (AdipoR3) are identified as the receptors for adiponectin (4). CTRP6 regulates adipocyte proliferation and differentiation as well as lipogenesis in myoblasts *via* AdipoR1 (5, 6). CTRP9 protects against acute cardiac damage and high glucose-induced endothelial oxidative damage, and inhibits macrophage-mediated inflammatory response against oxidized LDL *via* AdipoR1 (7–9). Thus, some CTRP family members share AdipoR1 and AdipoR2 as the common receptor, although complete ligand-receptor relationship has not been elucidated yet.

Adiponectin is suggested to inhibit dendritic cell (DC) activation, IFN-γ-producing helper T (Th1) cell differentiation and IL-17-producing helper T (Th17) cell differentiation *via* AdipoR1, AdipoR2 and unknown receptor(s) (10–12). AdipoRon, an agonist of AdipoR1 and AdipoR2 (13), treatment inhibited Th17 cell polarization in bleomycin-treated mice (14). However, other reports indicate that adiponectin induces DC activation and Th17 cell differentiation (15, 16). Furthermore, adiponectin activates the complement classical pathway through binding to C1q, whereas it inhibits the complement alternative pathway through binding to factor H (17, 18). CTRP6 inhibits the complement alternative pathway by competing C3(H_2_O) binding to factor B (19). CTRP6 also inhibits the lectin pathway (20). On the other hand, CTRP3 does not affect the complement system (21). Thus, the roles of each CTRP family member in the immune system seem very complex and further investigation is required to elucidate the mechanisms.

CTRP3 is implicated in the development of myocardiac dysfunction, inflammatory bowel diseases, severe acute pancreatitis and chronic kidney diseases (22–25). CTRP3 also functions as an antagonist for LPS, and modulates anti-inflammatory functions of monocytes, macrophages, adipocytes and fibroblasts (26–31). We identified AdipoR2, but not AdipoR1 nor AdipoR3, as a functional CTRP3 receptor and showed that CTRP3 regulates chondrocyte proliferation *via* this receptor (32). Previously, we showed that CTRP3 is highly expressed in rheumatoid arthritis (RA) models (19, 33), and that the development of collagen-induced arthritis (CIA) is exacerbated in *C1qtnf3* ^–/–^ mice in an complement-independent mechanism (21). In *C1qtnf3* ^–/–^ mice, type 2 collagen-specific antibody production and inflammatory cytokine production in joints are augmented, although CTRP3 does not directly affect cytokine production from neutrophils and synovial cells (21), leaving the anti-inflammatory mechanism of CTRP3 still obscure.

In this report, we investigated involvement of CTRP3 in the development of Th17 cells, because CTRP3 shares AdipoR2 with adiponectin, which is suggested to be involved in the differentiation of Th17 cells (10, 11, 34). Furthermore, we examined the effect of *C1qtnf3*-deficiency on the development of experimental autoimmune encephalomyelitis (EAE), in which Th17 cells play a crucial role (35). We found that CTRP3 suppresses Th17 cell differentiation *via* AdipoR2. We showed that Th17 cell differentiation is enhanced in *C1qtnf3* ^–/–^ mice and the development of EAE is exacerbated in these mutant mice. Interestingly, however, Th1 cell differentiation and DC function were intact in *C1qtnf3* ^–/–^ mice.

## Materials and Methods

### Ethics Statement

All experiments were approved by the Animal Care and Use Committee of Tokyo University of Science and Kansai Medical University, and were conducted according to the institutional ethical guidelines for animal experiments and safety guidelines for gene manipulation experiments.

### Mice

*C1qtnf3* ^–/–^ mice (B6-*C1qtnf3^tm1Yiw^*) were previously generated using C57BL/6N derived ES cells (21). C57BL/6J WT mice were purchased from Japan SLC (Japan), myelin oligodendrocyte glycoprotein (MOG) peptide, MOG_35-55_-specific TCR transgenic mice (2D2 mice: C57BL/6J-Tg(Tcra2D2, Tcrb2D2)1Kuch/J) were used for antigen-specific T cell recall response (36). We used 8-12 week-old mice of the same sex in all experiments. Mice were kept under specific pathogen–free conditions with 8:00-20:00 lighting cycle in clean rooms at the Center for Animal Disease Models, Research Institute for Biomedical Sciences, Tokyo University of Science (TUS). Mice were fed with γ-ray sterilized normal F1 diet (Funabashi Farm, Japan) and acidified tap water (pH 2.5, with 0.002 N HCl).

### Real-Time PCR

Total RNA was extracted using GenElute Mammalian Total RNA Miniprep Kit (Sigma-Aldrich, St. Louis, MO, USA) or Sepasol-RNA I Super (Nacalai tesque, Kyoto, Japan) and was reverse transcribed using High-Capacity cDNA Reverse Transcription Kit (Applied Biosystems, Foster City, CA, USA). We performed real-time RT-PCR using SYBR Green qPCR kit (Takara, Kyoto, Japan) and iCyclerSystem (Bio-Rad, Hercules, CA, USA) with a set of primers. The primer sequences were follows: *Gapdh*: 5’-TTCACCACCATGGAGAAGGC-3’ and 5’-GGCATGGACTGTGGTCATGA-3’. *C1qtnf3*: 5’-CTTCAGCATGTACAGCTATG-3’ and 5’-GTTGCCCATTCTTAGCCAGACT-3’. *Adipor1*: 5’-AGACAAGAGCAGGAGTGTTCCT-3’ and 5’-GGTGATGTACATCACAGCCATG-3’. *Adipor2*: 5’-GGACACATCTCCTAGGTTGTGT-3’ and 5’-TGGCAGTACACCGTGTGGAAGA-3’. *Ampka*: 5’-ACCTGAGAACGTCCTGCTTTG-3’ and 5’-GAAATGACTTCTGGTGCGGC-3’. *Ppara*: 5’- TGCAGCCTCAGCCAAGTTGAA-3’ and 5’- TCCCGAACTTGACCAGCCA-3’. *Rorc*: 5’- GACCCACACCTCACAAATTGA -3’ and 5’- AGTAGGCCACATTACACTGCT -3’. *Stat3*: 5’- CTTGTCTACCTCTACCCCGACAT-3’ and 5’- GATCCATGTCAAACGTGAGCG-3’. *Mtor*: 5’-TGTGAACGGAACATACGACC-3’ and 5’-TTGCTTGCCCATCAGAGTCAG-3’. According to delta-delta Ct method, the relative expression levels of target gene was normalized by a housekeeping gene (*Gapdh*).

### Cell Isolation

For real-time PCR analyses, T cells and B cells were purified from the spleen by anti-mouse CD90.2 and anti-mouse B220 microbeads (Miltenyi Biotec, Gladbach, Germany), respectively, and neutrophils were purified from bone marrow by anti-mouse Ly-6G microbeads, and monocytes were purified from bone marrow by anti-mouse Ly-6C microbeads (Miltenyi Biotec, Gladbach, Germany).

### T Cell Differentiation Assay

Naïve T cells were purified from spleen by naïve CD4^+^ T cell isolation kit (Miltenyi Biotec, Gladbach, Germany) using an AutoMACS (Miltenyi Biotec, Gladbach, Germany). Then, naïve T cells were cultured under Th17 cell-polarizing conditions; naïve T cells were cultured in 10 μg/ml anti-CD3 antibody (Ab) (clone 145-2C11; BioLegend, San Diego, CA, USA)-coated plates (IWAKI, Shizuoka, Japan) with 1 μg/ml anti-CD28 Ab (clone 37.51; BioLegend, San Diego, CA, USA), 10 μg/ml anti-IFN-γ Ab (clone R4-6A2; BioLegend, San Diego, CA, USA), 10 μg/ml anti-IL-4 Ab (clone 11B11; BioLegend, San Diego, CA, USA), 5 ng/ml recombinant human TGF-β (PeproTech, London, UK), 20 ng/ml recombinant murine IL-6 (PeproTech, London, UK) and 10 ng/ml recombinant murine IL-1β (PeproTech, London, UK) in X-VIVO 20 medium (Lonza, Switzerland) and were stimulated with/without recombinant human CTRP3 (Aviscera Bioscience, Santa Clara, CA, USA) or AdipoRon (Cayman Chemical Company, Ann Arbor, MI, USA) in absence or presence of AdipoR1 blocker (Alpha-Diagnostic International, USA) or AdipoR2 blocker (Alpha-Diagnostic International, USA).

Th1 cells were differentiated from naïve CD4^+^ T cells under Th1 cell-polarizing culture conditions; cells were cultured in 10 μg/ml anti-CD3 Ab -coated plates with 4 μg/ml anti-CD28 Ab, 10 μg/ml anti-IL-4 Ab, 5 ng/ml recombinant murine IL-12 (PeproTech, London, UK) in RPMI 1640 medium (Wako, Osaka, Japan) containing 10% FBS were stimulated with/without recombinant human CTRP3.

Th2 cells were differentiated from naïve CD4^+^ T cells under Th2 cell-polarizing culture conditions; cells were cultured in 10 μg/ml anti-CD3 Ab-coated plates with 4 μg/ml anti-CD28 Ab, 20 μg/ml anti-IFN-γ Ab, and 20 ng/ml recombinant murine IL-4 (PeproTech, London, UK) in RPMI 1640 medium containing 10% FBS.

We defined Th0 cells for naïve CD4^+^ T cells cultured in 10 μg/ml anti-CD3 Ab -coated plates with 4 μg/ml anti-CD28 Ab (clone 37.51; BioLegend, San Diego, CA, USA) in RPMI 1640 medium containing 10% FBS.

### Flow Cytometric Analysis

T cells were stimulated with 50 ng/ml PMA (Sigma-Aldrich, St. Louis, MO, USA), 500 ng/ml ionomycin (Sigma-Aldrich, St. Louis, MO, USA) and 2 μM monensin (Sigma-Aldrich, St. Louis, MO, USA) for 4 h. Cells were treated with an anti-mouse CD16/CD32 monoclonal Ab (2.4G2, purified from hybridoma culture supernatant) in a blocking buffer (HBSS containing 2% FCS and 0.1% sodium azide) to block Fc receptor binding, and then stained with Abs against mouse IL-17A (TC11-18H10.1), IFN-γ (XMG1.2) and CD4 (RM4-5) from Biolegend (San Diego, CA, USA) at 4°C for 30 min according to our standard procedures (37). Flow cytometry was carried out using a FACS Canto II cytometer and analyzed by either CellQuest (Becton Dickinson, San Jose, CA, USA) or FlowJo software (Tree Star, Ashland, OR, USA).

Bone marrow-derived dendritic cells (BMDCs) were treated with an anti-mouse CD16/CD32 Ab (2.4G2) in a blocking buffer (HBSS containing 2% FCS and 0.1% sodium azide) to block Fc receptor binding, and the then stained with Abs against mouse CD11c (N418), mouse CD40 (3/23), mouse CD80 (16-10A1) and CD86 (GL-1) from Biolegend (San Diego, CA, USA) at 4°C for 30 min according to our standard procedures (38).

### DC Activation Assay

According to a previous report (38), BMDCs were differentiated from bone marrow cells (BMCs) in femurs and tibiae by GM-CSF stimulation. Briefly, BMCs (5 x 10^4^ cells/well) were cultured in 96-well plates under 100 μl RPMI 1640 medium containing 10% FBS supplemented with 20 ng/ml mouse GM-CSF (PeproTech, London, UK), in the absence or presence of recombinant human CTRP3, and a half volume medium was changed by adding the same volume of the same medium at day 3 and day 6. At day 8, cell proliferation was measured by using the Cell Counting Kit-8 (Dojindo Laboratories, Kumamoto, Japan) and absorbance at 450 nm was measured using a microplate reader (MTP-300, Corona, Ibaragi, Japan).

For activation assay, BMCs (3 x 10^6^ cells/well) were cultured in 100-mm dish (IWAKI, Shizuoka, Japan) under 10 ml of RPMI 1640 medium containing 10% FBS supplemented with 20 ng/ml mouse GM-CSF, and a half volume medium was changed by adding the same volume of the same medium at day 3 and day 6. At day 8, the level of expression of maturation markers; CD11c, CD40, CD80 and CD86 was detected by flowcytometric analysis. To measure cytokine production, BMDCs were stimulated with zymosan (40 and 80 μg/ml; Sigma-Aldrich, St. Louis, MO, USA) or LPS (10 and 20 μg/ml; Sigma-Aldrich, St. Louis, MO, USA) with/without recombinant human CTRP3.

### ELISA

IL-17A, IFN-γ, IL-6 and TNF-α in culture supernatants were measured using mouse IL-17 ELISA MAX standard (R&D Systems, Minneapolis, MN, USA), mouse IFN-γ ELISA MAX standard (R&D Systems, Minneapolis, MN, USA), mouse IL-6 ELISA MAX standard (R&D Systems, Minneapolis, MN, USA) and mouse TNF-α ELISA MAX standard (R&D Systems, Minneapolis, MN, USA), respectively.

### EAE Induction

The myelin-oligodendrocyte glycoprotein (MOG)_35–55_ peptide (MEVGWYRSPFSRVVHLYRNGK) was synthesized by Scrum (Tokyo, Japan), and 20 mg of the peptide was dissolved in 1 ml of DMSO. To prepare MOG/CFA emulsion, 100 μl of 3 mg/ml MOG_35–55_ peptide in PBS was emulsified with 100 μl of 5 mg/ml *M. tuberculosis* (Difco, Detroit, MI, USA) in IFA (Thermo Scientific, MA, USA). Mice were immunized s.c. in flanks on day 0 with 200 μl of MOG/CFA emulsion. On day 7, mice were given a booster injection s.c. in flanks with the same amount of MOG/CFA (19). We evaluated the development of EAE macroscopically. The severity score was graded as follows: 0, no change; 0.5, partially limp tail; 1, paralyzed tail; 2, hind limp paresis; 2.5, one hind limb paralyzed; 3, both hind limbs paralyzed; 3.5, hind limbs paralyzed and weakness in forelimbs; 4, forelimbs paralyzed (39).

To measure the population of infiltrated IL-17^+^ T cells and IFN-γ^+^ T cells into the spinal cords at day 21 after immunization, mice under anesthesia were perfused with PBS to rinse blood off from the spinal cords through the intracardiac route using a peristaltic pump set (ATTO, Tokyo, Japan). The entire spinal columns were removed by gross dissection and spinal cords were ejected by a flush with a syringe attached in an 18-gauge needle, and then, spinal cords were incubated with 200 U/ml collagenase from *Clostridium histolyticum* (Sigma-Aldrich, St. Louis, MO, USA) in HBSS for 30 min at 37°C. The cell suspension of the spinal cords in 30% Percoll (Sigma-Aldrich, St. Louis, MO, USA) was overlaid on 70% Percoll in a 15 ml polypropylene tube and centrifuged at 2,200 rpm for 20 min at room temperature. Cells were collected from the interface between 30% and 70% Percoll and the population of IL-17^+^ T cells and IFN-γ^+^ T cells were analyzed by flow cytometry according to the standard techniques.

### Histological Analysis

According to the previous report (40), sections of the spinal cord were prepared at day 28 after immunization with MOG/CFA. Serial sections (5 μm) were stained with hematoxylin and eosin (H&E) or luxol fast blue (LFB, Nacalai tesque, Kyoto, Japan), and images were taken using a slide scanner (NanoZoomer, Hamamatsu Photonics, Shizuoka, Japan).

### T Cell Recall Response

At day 7 after immunization with MOG/CFA, lymph node (LN) cells (5 x 10^5^ cells/well) in 96-well plate were cultured in the absence or presence of 25 or 50 μg/ml MOG_35–55_ peptide for 72 h, followed by [^3^H]thymidine (0.25 μCi/ml) (Amersham, Amersham, UK) for 6 h. Then, LN cells were harvested with Micro 96 cell harvester (Skatron, Lier, Norway) and acid-insoluble radioactivity was measured with Micro Beta (Pharmacia Biotech, Piscataway, NJ, USA). IL-17 and IFN-γ concentrations in the culture supernatants from the proliferation assay after 66 h were measured by ELISA.

### Co-Culture of T Cells and BMDCs

T cells were purified from the spleen of 2D2 mice using anti-mouse Thy1.2 Ab-conjugated magnetic beads (#130-121-278, Miltenyi Biotec, Gladbach, Germany) and an AutoMACS (Miltenyi Biotec, Gladbach, Germany). The T cells were co-cultured with BMDCs in the presence of 5 μg/ml MOG_35-55_ peptide for 5 days. IL-17 and IFN-γ concentrations in the culture supernatants were measured by ELISA.

### Statistics

Unless otherwise specified, all results are shown as averages and SEM. Student’s *t*-test was used to evaluate the statistical significance of the results, except that χ^2^-test was used to evaluate incidence of EAE and Mann-Whitney *U*-test was used to evaluate severity score and mean maximum score of EAE. *p* values of < 0.05 were considered to be statistically significant.

## Results

### CTRP3 Suppresses Th17 Cell Differentiation *via* AdipoR2 Receptor

**Figure 1:**
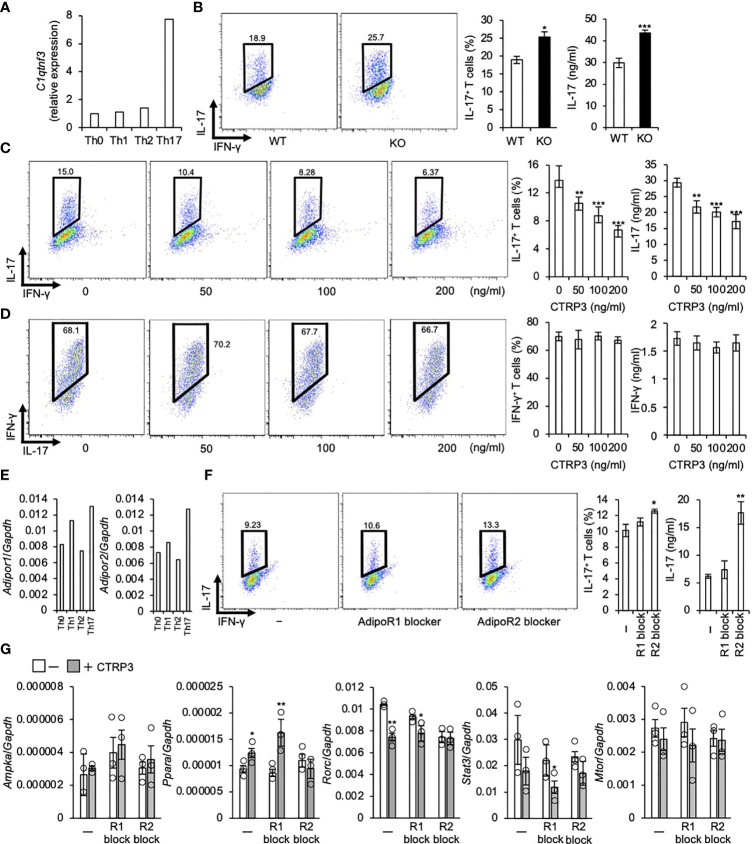
CTRP3 inhibits the differentiation of Th17 cells in an autocrine manner. **(A)** The relative expression of *C1qtnf3* mRNA in different Th subsets was determined by real-time PCR, and the expression levels are shown relative to that of Th0 cells. Th0, Th1, Th2 and Th17 cells were prepared as described in the *Materials and Methods* section. **(B)** WT or *C1qtnf3* ^–/–^ (KO) naïve CD4^+^ T cells were cultured under Th17-polarizing conditions for 4 days (n = 4 each). Intracellular IL-17 expression was estimated by flow cytometry after PMA/ionomycin stimulation. The numbers in each panel indicates percentage of IL-17^+^CD4^+^ T cells in total CD4^+^ T cells (2 left panels). The content of IL-17^+^CD4^+^ T cells in total CD4^+^ T cells (%) (center), and IL-17 concentrations (ng/ml) in culture supernatant determined by ELISA (right). Average and SEM are shown. *p < 0.05, ***p < 0.001. Student’s *t*-test. **(C)** *C1qtnf3* ^–/–^ naïve CD4^+^ T cells were cultured under Th17-polarizing conditions in the absence or presence of recombinant CTRP3 (50, 100 and 200 ng/ml) for 4 days (n = 4 wells each). Intracellular IL-17 expression was estimated by flow cytometry after PMA/ionomycin stimulation. The number in each panel indicates the percentage of IL-17^+^CD4^+^ T cells (left), and the proportion of IL-17^+^CD4^+^ T cells is shown in the center panel. IL-17 concentration in the culture supernatant was determined by ELISA (right). Average and SEM are shown. **p < 0.01, ***p < 0.001. Student’s *t*-test. **(D)** *C1qtnf3* ^–/–^naïve CD4^+^ T cells were cultured under Th1-polarizing conditions for 3 days (n = 4 wells each). Intracellular IFN-γ expression was evaluated by flow cytometry after PMA/ionomycin stimulation. The number in each panel indicates the percentage of IFN-γ ^+^CD4^+^ T cells in total CD4 T cells (left). The population of IFN-γ ^+^CD4^+^ T cells (center). IFN-γ concentration in the culture supernatant determined by ELISA (right). Average and SEM are shown. Student’s *t*-test. **(E)** The expression of *Adipor1* and *Adipor2* mRNA in different Th subsets was determined by real-time PCR and relative expression levels to that in Th0 cells are shown. **(F)** *C1qtnf3* ^–/–^ naïve CD4^+^ T cells were cultured with recombinant CTRP3 (200 ng/ml) under Th17-polarizing conditions in the absence (-) or presence of AdipoR1 blocker (R1 block, 10 μg/ml) or AdipoR2 blocker (R2 block, 10 μg/ml) for 4 days (n = 4 each). Intracellular IL-17 expression was evaluated by flow cytometry after PMA/ionomycin stimulation. The number in each panel indicates percentage of IL-17^+^CD4^+^ T cells (left). The IL-17^+^CD4^+^ T cell content (center). IL-17 concentration in the culture supernatant determined by ELISA (right). Average and SEM are shown. *p < 0.05, **p < 0.01. Student’s *t*-test. **(G)** The effect of AdipoR1 blocker and AdipoR2 blocker on the regulatory effects of CTRP3 on Th17 cell gene expression was examined. *In vitro* differentiated *C1qtnf3* ^–/–^ Th17 cells (1 x 10^5^ cells in 200 μl/96 well) were treated with CTRP3 (200 ng/ml) in the presence or absence of AdipoR1 blocker (10 μg/ml, R1 block) or AdipoR2 blocker (10 μg/ml, R2 block), and the expression of *Ampka*, *Ppara*, *Rorc*, *Stat3* and *Mtor* mRNA was determined by real-time PCR. The relative expression levels to that of *Gapdh* are shown. These data are the average from three independent experiments. Average and SEM are shown. *p < 0.05, **p < 0.01. Student’s *t*-test. All data were reproduced in another independent experiment with similar results.

Because *C1qtnf3* was highly expressed in Th17 cells (**Figure 1A**), we assessed the role of CTRP3 in Th17 cell differentiation. We found that *C1qtnf3* ^–/–^ naïve T cells differentiated to Th17 cells more efficiently compared with naïve T cells from WT mice under Th17 cell-polarizing culture conditions (**Figure 1B**). Furthermore, exogenous CTRP3 suppressed Th17 cell differentiation in a dose-dependent manner (**Figure 1C**). However, CTRP3 did not affect Th1 cell differentiation at all under Th1 cell-polarizing culture conditions (**Figure 1D**). These results suggest that CTRP3 specifically inhibits Th17 cell differentiation, but not Th1 cell differentiation, in an autocrine manner.

Consistent with a previous report (15), *Adipor1* and *Adipor2* mRNA were highly expressed in Th17 cells compared to naïve T cells. *Adipor1* mRNA levels in Th1 cells were higher than those in naïve T cells, while *Adipor2* mRNA levels were comparable to those in naïve T cells (**Figure 1E**). Previously, we identified that AdipoR2, but not AdipoR1, is a functional CTRP3 receptor on chondrogenic cells using siRNAs and blockers for AdipoR1 and AdipoR2 (32). Then, we examined whether or not CTRP3 inhibits Th17 cell differentiation *via* AdipoR2 using a peptide blocker. We found that AdipoR2 blocker, but not AdipoR1 blocker, abolished Th17-differentiation-inhibitory effect of CTRP3 (**Figure 1F**). Next, we analyzed *Ampka* and *Ppara* mRNA expression, because AdipoR1 activates AMPK pathway and AdipoR2 activates PPARα pathway, respectively (41). We found that CTRP3 increased *Ppara*, but not *Ampka*, mRNA expression in T cells under Th17 cell-polarizing culture conditions (**Figure 1G**). AdipoR1 blocker did not affect *Ampka* mRNA expression, while AdipoR2 blocker abolished the increment of *Ppara* mRNA expression (**Figure 1G**). Furthermore, CTRP3 decreased *Rorc* and *Stat3* mRNA expression. The decreased expression was abolished by AdipoR2 blocker, but not AdipoR1. CTRP3 did not significantly affect *Mtor* mRNA expression (**Figure 1G**). Thus, AdipoR2 is the functional receptor for CTRP3 on naïve T cells to inhibit Th17 cell differentiation.

**Figure 2:**
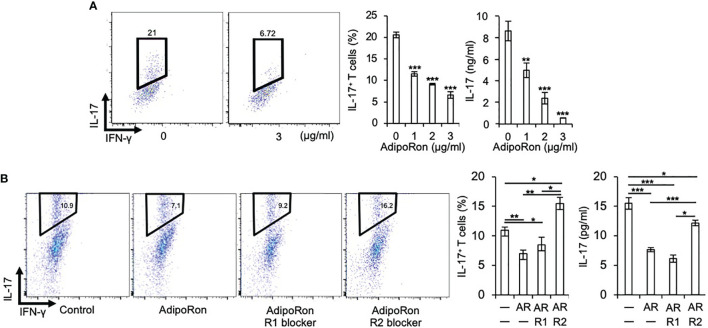
AdipoRon suppresses Th17 cell differentiation *via* AdipoR2. **(A)** *C1qtnf3* ^–/–^ naïve CD4^+^ T cells were cultured under Th17-polarizing conditions in the absence or presence of AdipoRon (0, 1, 2 and 3 μg/ml) for 5 days (n = 4 wells each). Intracellular IL-17 expression was estimated by flow cytometry after PMA/ionomycin stimulation. The number in each panel indicates percentage of IL-17^+^CD4^+^ T cells (2 left panels). The population of IL-17^+^CD4^+^ T cells (center). IL-17 concentrations in culture supernatant determined by ELISA (right). Average and SEM are shown. **p < 0.01 and ***p < 0.001. Student’s *t*-test. **(B)** *C1qtnf3* ^–/–^ naïve CD4^+^ T cells were cultured under Th17-polarizing conditions in the absence or presence of AdipoRon (1 μg/ml, Ron) for 4 days (n = 3 wells each), and the effect of AdipoR1 (10 μg/ml, R1) or AdipoR2 blocker (10 μg/ml, R2) on the Th17 cell differentiation was examined. Intracellular IL-17 expression was estimated by flow cytometry after PMA/ionomycin stimulation. The Number in each panel indicates percentage of IL-17^+^CD4^+^ T cells (4 left panels). The population of IL-17^+^CD4^+^ T cells (center). IL-17 concentrations in culture supernatant determined by ELISA (right). Average and SEM are shown. *p < 0.05, **p < 0.01 and ***p < 0.001. Student’s *t*-test. All data were reproduced in another independent experiment with similar results.

Furthermore, we assessed the role of AdipoR1 and AdipoR2 in the Th17 cell differentiation inhibition by AdipoRon. We found that AdipoRon suppressed Th17 cell differentiation of naive T cells from *C1qtnf3* ^–/–^ mice, in which no endogenous CTRP3 was present (**Figure 2A**). AdipoR2 blocker, but not AdipoR1 blocker, abolished the Th17-differentiation-inhibitory effect of AdipoRon (**Figure 2B**). These results indicate that AdipoR2 mediates the suppression of Th17 cell differentiation.

### Development of EAE Is Exacerbated in *C1qtnf3* ^–/–^ Mice Associated With an Increase of Th17 Cells

**Figure 3:**
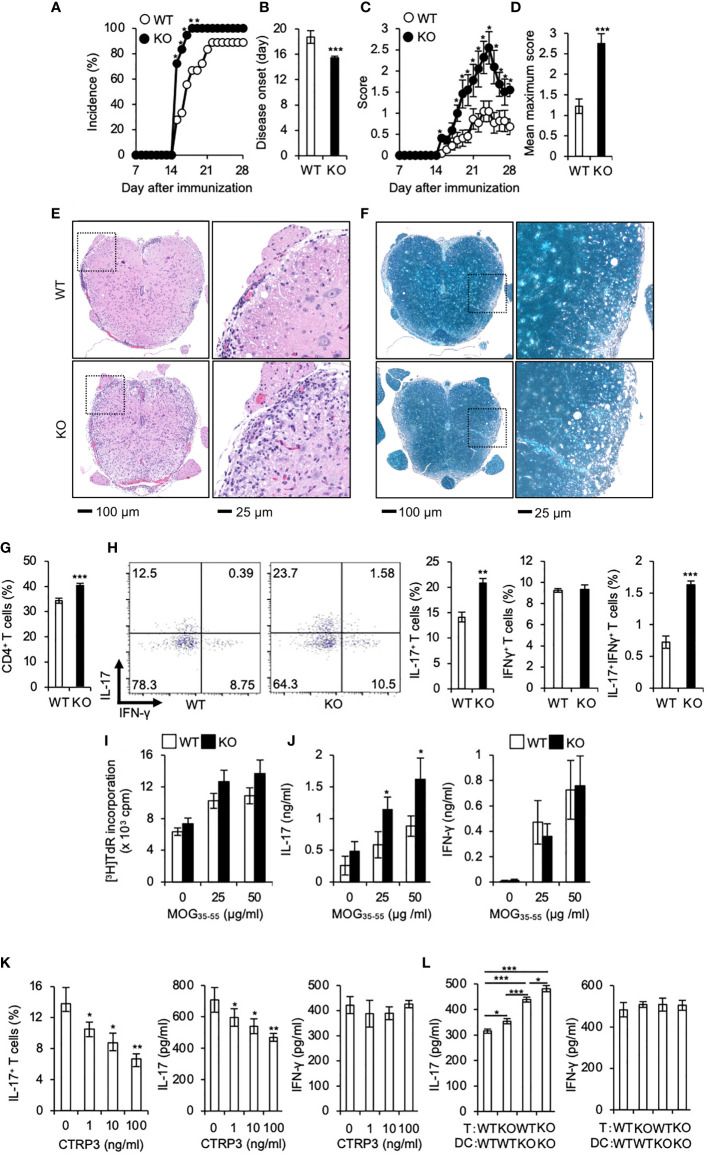
Development of EAE is exacerbated in *C1qtnf3* ^–/–^ mice associated with an augmented Th17 cell differentiation. **(A–D)** Incidence of affected mice **(A)**, onset time **(B)**, disease score time course **(C)** and mean maximum disease score **(D)** are shown after induction of EAE (11 mice for each group). Average and SEM are shown. *p < 0.05 and ***p < 0.001. χ^2^-test. Mann-Whitney *U*-test. Student’s *t*-test. **(E, F)** Tissue sections of the spinal cord were stained with H&E **(E)** and LFB **(F)** at day 28 after induction of EAE. **(G, H)** CD4^+^ T cells **(G)**, CD4^+^ IL-17^+^ and CD4^+^ IFN-γ^+^ T cells **(H)** in the whole spinal cord from WT and *C1qtnf3* ^–/–^ (KO) mice at day 21 after immunization were determined by flow cytometry after PMA/ionomycin stimulation (WT, KO: n = 5 each). The numbers in each panel indicate the percentage of IL-17^+^, IFN-γ^+^, and IL-17^+^IFN-γ^+^ T cells (**H**, 2 left panels). The population of IL-17-single positive, IFN-γ-single positive and IL-17-, IFN-γ-double positive T cells (H, 3 right panels). Average and SEM are shown. ***p < 0.001. Student’s *t*-test. **(I, J)** T cell recall response against the MOG peptide. Seven days after MOG/CFA-immunization, LN cells were isolated from WT and *C1qtnf3* ^–/–^ (KO) mice and re-stimulated with MOG_35-55_ (0, 25, 50 μg/ml) for 72 h (n = 5 each). Then, the proliferative response was measured by [^3^H]TdR incorporation **(I)**. IL-17 (**J**, left) and IFN-γ (**J**, right) concentrations in the culture supernatant were determined by ELISA. Average and SEM are shown. *p < 0.05. Student’s *t*-test. **(K)** *C1qtnf3* ^–/–^ 2D2 T cells and *C1qtnf3* ^-/-^ BMDCs were cultured with 5 μg/ml MOG_35-55_ in the presence or absence of CTRP3 (0, 1, 10, 100 ng/ml) for 5 days, and Th17 cell content (left), and IL-17 (center) and IFN-γ (right) concentrations in the culture supernatant were determined (n = 4 wells each). Average and SEM are shown. *p < 0.05 and **p < 0.01. Student’s *t*-test. **(L)** WT or *C1qtnf3* ^-/-^ 2D2 T cells were cultured with WT or *C1qtnf3* ^-/-^ BMDCs in the presence of 5 μg/ml MOG_35-55_ for 5 days (n = 3 each), and IL-17 (left) and IFN-γ (right) concentrations in the culture supernatant were determined. Average and SEM are shown. *p < 0.05, **p < 0.01 and ***p < 0.001. Student’s *t*-test. All data were reproduced in another independent experiment with similar results.

Th17 cells play a pivotal role in the pathogenesis of multiple sclerosis (MS) (42, 43) and the mouse model EAE (35, 44). Because CTRP3 regulates the differentiation of Th17 cells, we examined the role of CTRP3 in the pathogenesis of EAE. The incidence of EAE was increased in *C1qtnf3* ^–/–^ mice than WT mice (**Figure 3A**). The onset of paralysis in *C1qtnf3* ^–/–^ mice was earlier than WT mice (**Figure 3B**). Chronological disease scores and the maximum score in *C1qtnf3* ^–/–^ mice were markedly increased compared to WT mice (**Figures 3C, D**). The infiltration of immune cells and demyelination in spinal cords at the lumber level were more severe in *C1qtnf3* ^–/–^ mice (**Figures 3E, F**). Since infiltration of Th17 cells into central nervous system is required for the initiation of EAE (45, 46), we examined infiltration of Th17 cells in the spinal cord. We found that CD4^+^ T cell (**Figure 3G**) and CD4^+^IL-17^+^ T cell (Th17 cell) infiltration increased in *C1qtnf3* ^–/–^ mice compared to WT mice, but infiltrated CD4^+^IFN-γ ^+^ T cell (Th1 cell) population was comparable between WT and *C1qtnf3* ^–/–^ mice (**Figure 3H**).

Consistent with our previous report (21), T cell recall proliferative response against MOG peptide was comparable between WT mice and *C1qtnf3* ^–/–^ mice (**Figure 3I**). The culture supernatant of MOG-stimulated T cells from *C1qtnf3* ^–/–^ mice contained higher concentrations of IL-17, but not IFN-γ, compared to that of WT mice (**Figure 3J**). Furthermore, exogenous CTRP3 suppressed IL-17 production from MOG-specific 2D2 cells upon incubation with MOG peptide (**Figure 3K**). In contrast, IFN-γ production was not affected by CTRP3 at all.

**Figure 4:**
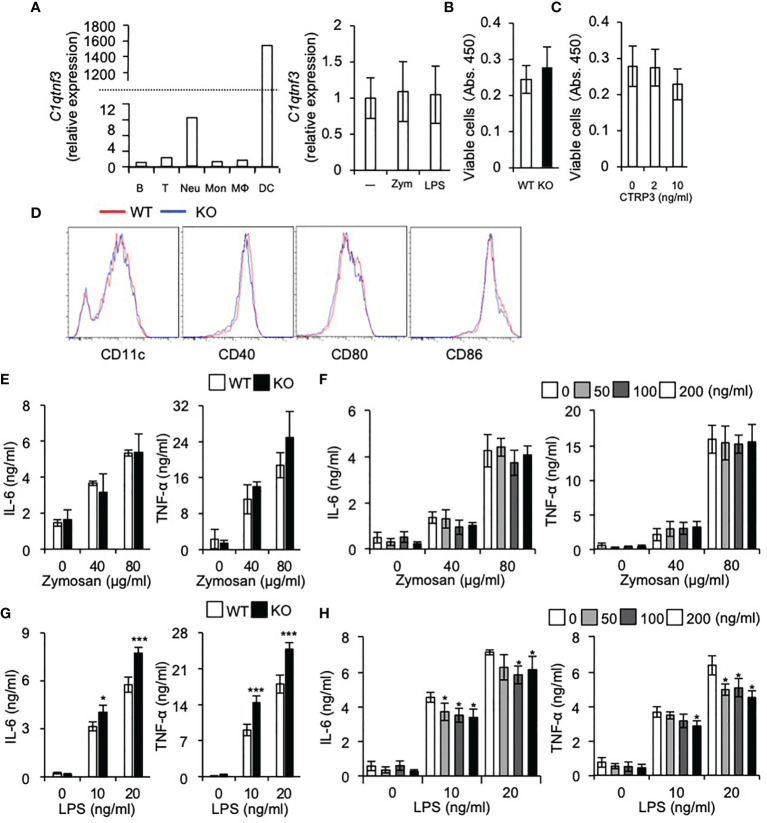
DC differentiation is normal in *C1qtnf3* ^-/-^ mice. **(A)** *C1qtnf3* mRNA in immune cells was determined by real-time PCR, and the relative expression levels to that in B cells are shown in left panel. *C1qtnf3* mRNA expression in DCs after stimulation with zymosan or LPS was determined by real-time PCR, and the relative expression levels to that in cells without stimulation (-) are shown in right panel. **(B, C)** Effect of CTRP3 on the differentiation and proliferation of BMDCs. BMDCs were differentiated from WT or *C1qtnf3* ^–/–^ (KO) BMCs **(B)**, or from *C1qtnf3* ^–/–^ BMCs in the presence of indicated concentrations of CTRP3 **(C)**. Then, viable cell number was counted at day 8 by using Cell Counting Kit-8. 4 wells for each. Average and SEM are shown. Student’s *t*-test. **(D)** WT or *C1qtnf3* ^-/-^ (KO) BMCs were cultured under BMDC-differentiation conditions, and the expression of CD11c, CD40, CD80 and CD88 was determined by flow cytometry at day 8. **(E)** IL-6 and TNF-α concentration in the culture supernatant of WT or *C1qtnf3* ^–/–^ (KO) BMDCs were measured by ELISA after stimulated with zymosan (0, 40 and 80 μg/ml). Four wells for each. Average and SEM are shown. Student’s *t*-test. **(F)** IL-6 and TNF-α concentration in the culture supernatant of *C1qtnf3* ^–/–^ BMDCs were measured by ELISA after stimulated with zymosan (0, 40 and 80 μg/ml) with/without CTRP3. Four wells for each. Average and SEM are shown. Student’s *t*-test. **(G)** IL-6 and TNF-α concentration in the culture supernatant of WT or *C1qtnf3* ^–/–^ (KO) BMDCs were measured by ELISA after stimulated with LPS (0, 10 and 20 ng/ml). Four wells for each. Average and SEM are shown. *p < 0.05, ***p < 0.001. Student’s *t*-test. **(H)** IL-6 and TNF-α concentration in the culture supernatant of *C1qtnf3* ^–/–^ BMDCs were measured by ELISA after stimulated with LPS (0, 10 and 20 ng/ml) with/without CTRP3. Four wells for each. Average and SEM are shown. *p < 0.05. Student’s *t*-test. All data were reproduced in another independent experiment with similar results.

Next, we assessed contribution of T cell-derived CTRP3 and DC-derived CTRP3 on the suppression of Th17 cell differentiation. MOG-specific TCR-expressing T cells were prepared from 2D2 and *C1qtnf3* ^–/–^ 2D2 mice and BMDCs were prepared from WT and *C1qtnf3* ^–/–^ mice, respectively, and they were cocultured in the presence of MOG_35-55_ for 5 days and IL-17 and IFN-γ production were measured. Higher concentration of IL-17 was detected in the culture supernatant in the co-culture of *C1qtnf3* ^–/–^ 2D2 T cells and WT BMDCs compared with that in WT T cell and WT DC coculture, indicating that T cell-derived CTRP3 suppressed Th17 cell differentiation in an autocrine manner. Furthermore, co-culture of WT 2D2 T cells with *C1qtnf3* ^–/–^ BMDCs also produced higher concentrations of IL-17 than the co-culture of WT 2D2 T cells and WT BMDCs, indicating that Th17 cell differentiation was also suppressed by the CTRP3 produced by DCs in a paracrine manner (**Figure 3L**). As CTRP3 was highly expressed in DCs than T cells (**Figure 4A**), IL-17 concentration in the co-culture of WT T cells and *C1qtnf3* ^–/–^ BMDCs was higher than that of *C1qtnf3* ^–/–^ T cells and WT BMDCs. These observations suggest that CTRP3 regulates the development of EAE by inhibiting Th17 cell differentiation in an autocrine and paracrine manner.

### DC Differentiation Is Normal in *C1qtnf3* ^–/–^ Mice

We found that *C1qtnf3* mRNA is highly expressed in DCs and neutrophils among other immune cells and stimulation of DCs with zymosan or LPS did not affect expression of *C1qtnf3* mRNA expression (**Figure 4A**). Then, we examined the effects of CTRP3 on *in vitro* differentiation of BMDCs from BMCs. Regarding this, we and other researchers showed that CTRP3 promotes proliferation of specific types of cells such as chondrogenic cells, endothelial cells and myoblasts (32, 47–53). We found that cell number of BMDCs from *C1qtnf3* ^–/–^ BMCs was similar to WT BMC-derived BMDCs (**Figure 4B**). Furthermore, treatment with exogenous CTRP3 did not affect the cell number of BMDCs *in vitro* (**Figure 4C**). The expression levels of maturation and activation markers on *C1qtnf3* ^–/–^ BMDCs were comparable with BMDCs from WT mice, indicating that CTRP3 does not affect BMDC differentiation (**Figure 4D**). Next, we stimulated BMDCs with zymosan and LPS, and examined cytokine production. IL-6 and TNF-α production from BMDCs by zymosan stimulation were comparable between WT and *C1qtnf3* ^–/–^ mouse-derived cells (**Figure 4E**). Furthermore, exogenous CTRP3 did not affect zymosan-induced IL-6 and TNF-α production in *C1qtnf3* ^–/–^ BMDCs (**Figure 4F**). On the other hand, cytokine productions by LPS stimulation were increased in *C1qtnf3* ^–/–^ BMDCs (**Figure 4G**). Exogenous CTRP3 suppressed LPS-induced IL-6 and TNF-α expression in *C1qtnf3* ^–/–^ BMDCs (**Figure 4H**). This is probably because CTRP3 antagonizes LPS binding to TLR4 by binding to LPS (26). These results suggest that CTRP3 is dispensable for DC maturation and activation.

## Discussion

Here, we showed that CTRP3 inhibits Th17 cell differentiation *via* AdipoR2 in autocrine and paracrine manner. On the other hand, CTRP3 did not affect Th1 cell differentiation and BMDC activation, although these cells also express AdipoR2. Development of EAE was exacerbated in *C1qtnf3* ^–/–^ mice associated with over-expansion of Th17 cells.

Adiponectin receptors AdipoR1 and AdipoR2 share 66.7% amino acid identity (4). These receptors have common and different biological functions. We and other researchers showed that AdipoR1 and AdipoR2 express on Th17 cells (15). Although the effect of adiponectin on Th17 cell differentiation remains controversial (10, 11, 15), we showed that CTRP3-mediated inhibition of Th17 cell differentiation was abolished by AdipoR2 blocker, but not AdipoR1 blocker. Furthermore, AdipoRon regulated Th17 cell differentiation *via* AdipoR2, but not AdipoR1. These results demonstrate that AdipoR2 has a crucial role in the regulation of Th17 cell differentiation.

**Figure 5:**
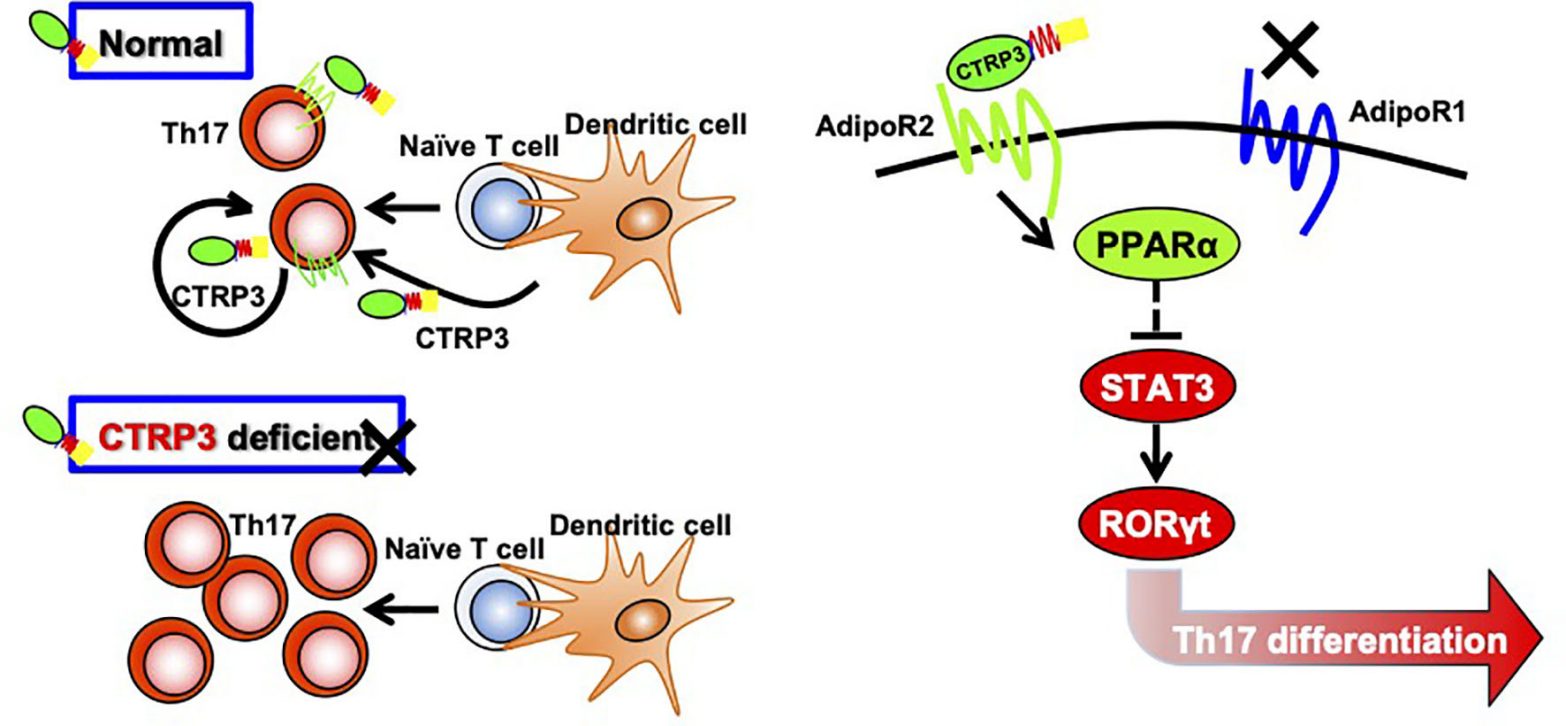
Regulation of Th17 cell differentiation by CTRP3 through AdipoR2. CTRP3 mainly expressed in dendritic cells and Th17 cells regulates Th17 cell differentiation through activation of AdipoR2, but not *via* AdipoR1. The CTRP3/AdipoR2 axis enhances PPARα expression and inhibits STAT3 and RORγt expression.

AdipoR1 and AdipoR2 activate different signaling pathways; AdipoR1 mediates AMPK signaling pathway whereas AdipoR2 mediates PPARα signaling pathway (41). We showed that CTRP3 increased *Ppara* mRNA expression and decreased *Rorc* and *Stat3* mRNA expression in Th17 cells. Transcription factor RORγt is a master regulator of Th17 cells (54), and STAT3 activation induces RORγt expression (55), while mTOR controls Th17 cell differentiation *via* regulation of RORγt activation (56). CTRP3-induced modification of gene expression was abolished by AdipoR2 blocker, but not AdipoR1 blocker. It was reported that PPARα suppresses Th17 cell differentiation *via* inhibition of the STAT3/RORγt signaling pathway (57). Furthermore, a PPARα agonist Fenofibrate suppresses Th17 cell differentiation, but not Th1 and Th2 cell differentiation, by reducing STAT3 activation (58). Thus, these findings suggest that the CTRP3/AdipoR2/PPARα axis suppresses Th17 cell differentiation *via* inhibition of the STAT3/RORγt signaling pathway (**Figure 5**).

It is well-known that complement C1q is a hetero-multimer of C1qA, C1qB and C1qC associated through the collagen-like domain (59). Likewise, adiponectin can form homo- and hetero-multimers (60), and CTRPs also form hetero-oligomers between different CTRPs, such as adiponectin/CTRP2, adiponectin/CTRP9, CTRP1/CTRP6 and CTRP2/CTRP7 hetero-oligomers (61, 62). A C1q-related factor (CRF, CTRP14) also forms hetero-oligomers with CTRP1, CTRP9, CTRP8 and CTRP10 (63). CTRP9A and CTRP9B, having 98% amino acid identify, form a CTRP9A/CTRP9B hetero complex (63). The biological functions and receptors of these CTRP hetero-oligomers remain to be elucidated. Thus, it is possible that CTRP3 forms oligomers with other CTRP family members and exerts unknown biological functions in AdipoR1/R2-expressing cells. Regarding this, it was reported that splicing variants of human CTRP3 form hetero-oligomers, which are more stable to proteolytic cleavage (64).

On the other hand, Ruiz et al. showed that AdipoR1 and AdipoR2 maintain membrane fluidity in several types of cells, although adiponectin is not required for this biological function of AdipoRs (65). Adiponectin, CTRP6 and CTRP9 share AdipoR1 as a common receptor, and adiponectin and CTRP3 share AdipoR2 (5, 6, 8, 32, 66). Thus, distinct CTRPs may exert a common or similar function (3), depending on cell types. Furthermore, Zha et al. reported that angiotensin II receptors (AT1 and AT2) form heterodimer with AdipoR1 and AdipoR2 and inhibit the biological action of AdipoRs in renal tubular epithelial cells (67). Thus, AdipoR signalings may also be regulated by antagonizing receptors, making the regulation of the CTRP-AdipoR system very complex.

Many apparently inconsistent results are reported related to the CTRP family ligand-receptor relationships and their functions. We showed in this report that CTRP3 inhibits specifically Th17, but not Th1, cell differentiation through AdipoR2-mediated mechanism using AdipoR-specific blocking peptides. We did not observe any effects of AdipoR1 inhibition on CTRP3-regulated Th17 cell differentiation. On the other hand, adiponectin inhibits both Th1 and Th17 cell differentiation *via* AdipoR1, AdipoR2 and unknown receptor(s) (10–12, 68). These observations are consistent with the idea that Th17 cell differentiation is inhibited *via* AdipoR2. However, recently Zhang et al. reported that Th17 cell differentiation is suppressed in AdipoR1-deficient mice (69), suggesting an enhancing role of AdipoR1 for the differentiation of Th17 cells. Although this apparently seems contradict with previous reports (11–13), this is probably because signaling through AdipoR2 is enhanced in the absence of AdipoR1. Actually, we found that AdipoRon, an agonist of AdipoR1 and AdipoR2, inhibits Th17 cell differentiation. The effect of AdipoRon on Th17 cell differentiation was abolished by AdipoR2 blocker, but not AdipoR1 blocker.

Similar to Th17 cells and chondrocytes, DCs also express AdipoR1 and AdipoR2. Previously, we reported that T cell recall response was normal in *C1qtnf3* ^–/–^ mice (21), and in this report, we showed that DC functions including proliferation and maturation are normal in CTRP3-deficient DCs. Consistent with our results, DC maturation and phagocytic capacity remain unaffected by the treatment with adiponectin (70). However, in this report, we found that IL-17 production was enhanced in a MOG-specific T cell-*C1qtnf3* ^–/–^ DC-coculture, suggesting DC function is also suppressed by CTRP3. Consistent with our observations, Tan et al. showed that adiponectin arrests DC function by blocking NF-κB activation *via* AdipoR1 and AdipoR2 (68). Kupchak et al. also showed that DC differentiation and maturation are enhanced through inhibition of AdipoR1 and AdipoR2 by TNF-α derived from GM-CSF-activated BMDCs (71). However, Jung et al. reported that adiponectin induces functional maturation of DCs independently from AdipoR1 and AdipoR2 and Th1 and Th17 cell differentiation are enhanced in coculture with adiponectin-primed DCs (16). Thus, AdipoR functions are still controversial depending on the cellular and experimental settings. Clearly, further investigation is necessary to elucidate the detailed mechanisms.

In this report, we have demonstrated that CTRP3 ameliorates development of EAE by inhibiting Th17 cell differentiation. We have already shown that CTRP3 ameliorates development of autoimmune arthritis (21). Furthermore, CTRP3 is implicated in the regulation of myocardiac dysfunction, inflammatory bowel disease, severe acute pancreatitis and chronic kidney diseases (21–25). Because involvement of Th17 cells is suggested in these diseases (72–75), it is possible that CTRP3 regulates excess inflammation in these diseases. Taken together, these results suggest that the CTRP3-AdipoR2 axis is a good target for the treatment of Th17 cell-mediated autoimmune diseases such as MS and RA.

## Data Availability Statement

The original contributions presented in the study are included in the article/supplementary material. Further inquiries can be directed to the corresponding author.

## Ethics Statement

The animal study was reviewed and approved by the Animal Care and Use Committee of Tokyo University of Science and Kansai Medical University.

## Author Contributions

MAM and YI designed research study and wrote the manuscript. MAM performed and analyzed most experiments with technical support from MM and TO, and H-HC. H-HC significantly contributed to the revision. All authors contributed to the article and approved the submitted version.

## Funding

This work was supported by JSPS KAKENHI (Grant No. JP17K14978, JP19K20681, JP21K06955, 20H04954 and JP21H02394).

## Conflict of Interest

The authors declare that the research was conducted in the absence of any commercial or financial relationships that could be construed as a potential conflict of interest.

## Publisher’s Note

All claims expressed in this article are solely those of the authors and do not necessarily represent those of their affiliated organizations, or those of the publisher, the editors and the reviewers. Any product that may be evaluated in this article, or claim that may be made by its manufacturer, is not guaranteed or endorsed by the publisher.
